# Impact of Prior Statin Therapy on In-Hospital Outcome of STEMI Patients Treated with Primary Percutaneous Coronary Intervention

**DOI:** 10.3390/jcm11185298

**Published:** 2022-09-08

**Authors:** Oreste Lanza, Nicola Cosentino, Claudia Lucci, Marta Resta, Mara Rubino, Valentina Milazzo, Monica De Metrio, Filippo Trombara, Jeness Campodonico, José Pablo Werba, Alice Bonomi, Giancarlo Marenzi

**Affiliations:** 1 Centro Cardiologico Monzino, I.R.C.C.S., 20138 Milan, Italy; 2 Cardiovascular Section, Department of Clinical Sciences and Community Health, University of Milan, 20122 Milan, Italy

**Keywords:** statins, ST-elevation myocardial infarction, primary percutaneous coronary intervention, in-hospital outcomes

## Abstract

**Background**: Prior statin therapy has a cardioprotective effect in patients undergoing elective or urgent percutaneous coronary intervention (PCI). However, data on patients with ST-elevation myocardial infarction (STEMI) undergoing primary PCI are still controversial. We retrospectively evaluated the effect of prior statin therapy on in-hospital clinical outcomes in consecutive STEMI patients undergoing primary PCI. **Methods:** A total of 1790 patients (mean age 67 ± 11 years, 1354 men) were included. At admission, all patients were interrogated about prior (>6 months) statin therapy. The primary endpoint of the study was the composite of in-hospital mortality, acute pulmonary edema, and cardiogenic shock in patients with or without prior statin therapy. **Results:** A total of 427 patients (24%) were on prior statin therapy. The incidence of the primary endpoint was similar in patients with or without prior statin therapy (15% vs. 16%; *p* = 0.38). However, at multivariate analysis, prior statin therapy was associated with a lower risk of the primary endpoint, after adjustment for major prognostic predictors (odds ratio 0.61 [95% CI 0.39–0.96]; *p* = 0.03). **Conclusions:** This study demonstrated that prior statin therapy is associated with a better in-hospital clinical outcome in patients with STEMI undergoing primary PCI compared to those without prior statin therapy.

## 1. Introduction

Primary percutaneous coronary intervention (pPCI) is the treatment of choice for patients presenting with ST-elevation myocardial infarction (STEMI) [1,2,3]. Timely pPCI is able to reduce infarct size and ventricular dysfunction, thus resulting in better clinical outcomes [4,5,6].

In patients with stable angina or in those presenting with non-ST-elevation acute coronary syndromes, statin pre-treatment reduces myocardial injury and improves prognosis, also thanks to their pleiotropic properties [7,8,9,10]. On the other hand, data on the clinical effects of statin pre-treatment in patients with STEMI treated by pPCI are controversial. Indeed, while experimental models showed a significant reduction in infarct size after myocardial reperfusion in models pre-treated with statins [11,12], clinical studies did not consistently find such a beneficial effect [13,14,15]. Although several explanations could be proposed, it can also be argued that in patients with STEMI, the time between statin loading, usually at hospital admission, and pPCI may be not enough for the achievement of statin-therapeutic circulating levels [15,16]. To this regard, it is worth noting that a 30% reduction in myocardial infarct size—as assessed by cardiac magnetic resonance—was observed in STEMI patients chronically treated with statins before the index hospitalization, as compared to those receiving statin at hospital admission [17,18]. Thus, whether or not this cardioprotective effect of prior statin therapy on infarct size reduction results in more favorable in-hospital clinical outcomes in STEMI patients undergoing percutaneous myocardial revascularization has not yet been fully clarified.

In this study, we evaluated in-hospital clinical outcomes in a consecutive cohort of patients with STEMI treated with pPCI, comparing patients on long-term statin with those without previous statin therapy.

## 2. Materials and Methods

### 2.1. Study Population

This retrospective study included all consecutive STEMI patients treated with pPCI between December 2010 and April 2021 at Centro Cardiologico Monzino (Milan, Italy). Patients undergoing cardiac surgery and those with uncertain statin compliance were excluded from the study. The study was approved by the Institutional Review Board of our institute (R519-CCM548).

### 2.2. Study Protocol

Demographic, clinical, biochemical, echocardiographic, and angiographic data were retrieved for all patients. Left ventricular ejection fraction (LVEF) was measured by echocardiogram in all patients soon after pPCI. All patients were interrogated about chronic therapy, including statins, and compliance before the index event. All patients (regardless of prior statin therapy), were treated with high-intensity statin therapy soon after hospital admission. 

The primary endpoint of the study was the combination of in-hospital mortality, non-fatal acute pulmonary edema, and cardiogenic shock in STEMI patients with and without prior statin therapy (any specific statin and any dose). We used this combined endpoint because cardiogenic shock and acute pulmonary edema are more closely associated with the extent of myocardial infarct size and acute ventricular dysfunction in STEMI. The secondary endpoint was LVEF value, measured soon after pPCI (within 24 h).

### 2.3. Statistical Analysis

A sample size of 1700 STEMI patients was calculated under the following assumptions: 25% of STEMI patients with prior statin therapy, 15% overall incidence of the primary endpoint, with an expected 12% and 18% incidence in patients with and without prior statin therapy, respectively. This sample size allowed for a 90% statistical power in assessing a significant difference (α error of 0.05) of the combined endpoint between the two study groups. 

Continuous variables are presented as mean ± SD, and they were compared with the *t*-test for independent samples. Variables not normally distributed are presented as median and interquartile ranges, and compared with the Mann–Whitney U test. Categorical variables were compared by the chi-square test or Fisher’s exact test, as appropriate.

The association between prior statin therapy and the study endpoints was assessed by logistic regression analysis. Analyses were adjusted for variables most closely associated with the primary endpoint (epidemiological approach): age, gender, prior acute myocardial infarction, diabetes mellitus, time-to-treatment, and STEMI location (anterior vs. non-anterior). Moreover, as patients with prior statin therapy were more frequently treated with beta-blockers and angiotensin-converting enzyme inhibitors/angiotensin II receptor blockers (ACEi/ARB) at hospital admission, these two variables were also added to the multivariable analysis. Results are presented as odds ratio (OR) with 95% confidence intervals (CI). 

Differences in the study endpoints among low-density lipoprotein-cholesterol (LDL-C) quartiles were assessed by ANOVA for continuous variables.

All tests were two-sided, and a *p* value < 0.05 was required for statistical significance. All calculations were computed with SAS software package (V. 9.4, SAS Institute Inc., Cary, NC, USA).

## 3. Results

A total of 1790 patients with STEMI treated with pPCI (mean age 67 ± 11 years, 1354 men) were included in the analysis. In particular, 427 patients (24%) were on prior statin treatment, with a treatment duration of >6 months before hospitalization in all patients. Table 1 reports the clinical characteristics of the patient’ groups according to whether they were on previous statin therapy or not. Patients on prior statin therapy were more likely to have cardiovascular risk factors and more likely to have experienced a prior cardiovascular event. Of note, admission total and LDL-C values were significantly lower in the statin patients, confirming the overall compliance to statin therapy. Table 2 shows the in-hospital clinical complications of the patient’ groups according to whether they were on previous statin therapy or not.

### Subsection

**Figure 1:**
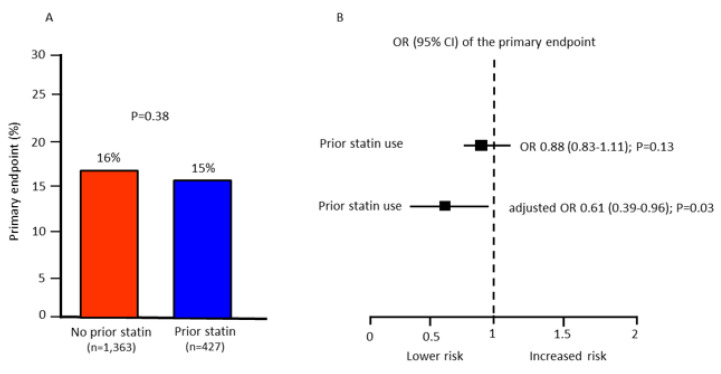
(**A**): Primary endpoint rate in patients with or without prior statin therapy. (**B**): crude and adjusted odds ratio (OR) and 95% confidence intervals (CI) of the primary endpoint associated with prior statin use. Odds ratio was adjusted for age, gender, prior acute myocardial infarction, diabetes mellitus, time-to-treatment, and STEMI location (anterior vs. non-anterior).

Patients on prior statin therapy showed a similar rate of the primary in-hospital endpoint compared to patients without prior statin therapy (15% vs. 16%; *p* = 0.38) (Figure 1A).

**Figure 2:**
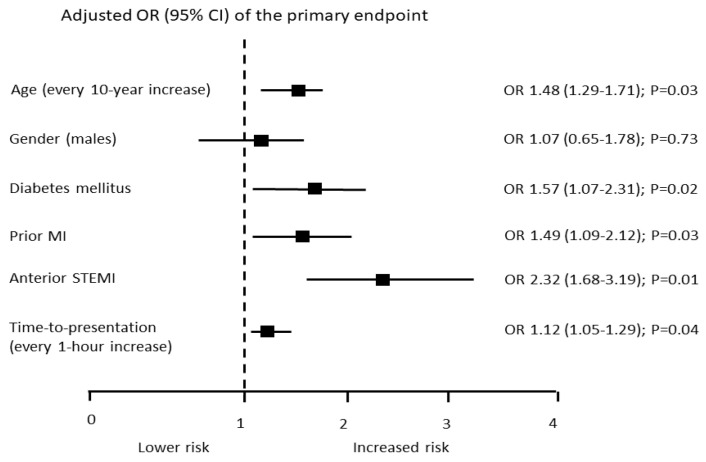
Odds ratio (OR) and 95% confidence interval (CI) of the primary endpoint of variables selected in our study, by using an epidemiological approach, as potential prognostic predictors in STEMI patients. Odds ratios were adjusted for each variable. MI = myocardial infarction.

At multivariate analysis, prior statin treatment was associated with a similar risk of the primary endpoint. However, when statin therapy was adjusted for the major predictors of the primary endpoint shown in Figure 2, it carried a lower risk of the endpoint (adjusted OR 0.61 [95% CI 0.39–0.96]; *p* = 0.03) (Figure 1B). Moreover, when prior therapy with beta-blockers and ACEi/ARB was added to the multivariable model, prior statin therapy was still associated with a lower risk of the primary endpoint (adjusted OR 0.72 [95% CI 0.45–0.98]; *p* = 0.04)

**Figure 3:**
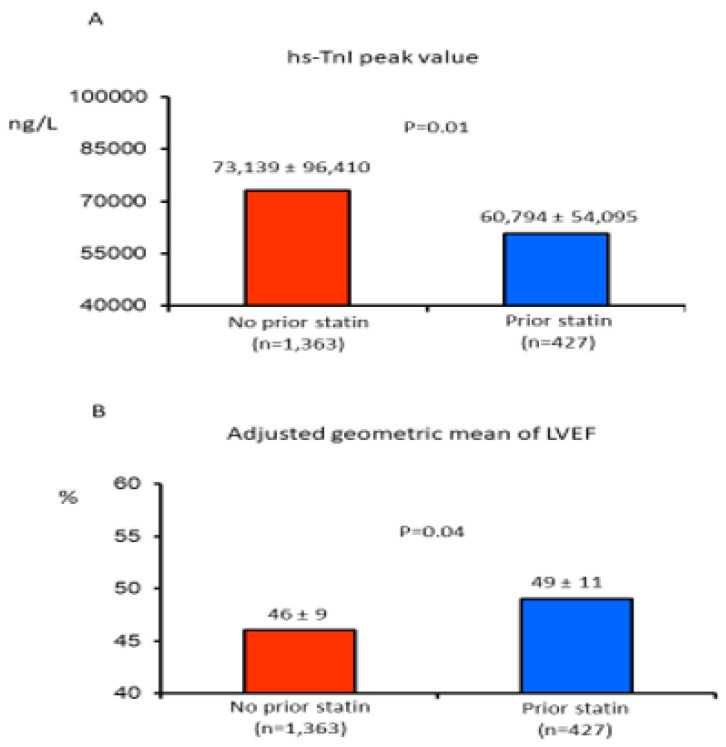
(**A**): high-sensitivity troponin I (hs-TnI) peak values in STEMI patients with and without prior statin therapy. (**B**): geometric mean of left ventricular ejection fraction (LVEF) adjusted for prior myocardial infarction in STEMI patients with or without prior statin therapy.

Patients on prior statin therapy showed a significantly (*p* < 0.01) lower troponin I peak value during hospitalization, as compared to those patients without prior statin therapy (Figure 3). At echocardiographic analysis, there were no differences in LVEF. However, given that patients on prior statin therapy were more likely to have had a previous myocardial infarction, when the geometric mean of the LVEF was adjusted for prior myocardial infarction, it was significantly higher in patients on prior statin therapy when compared to those without (Figure 3).

**Figure 4:**
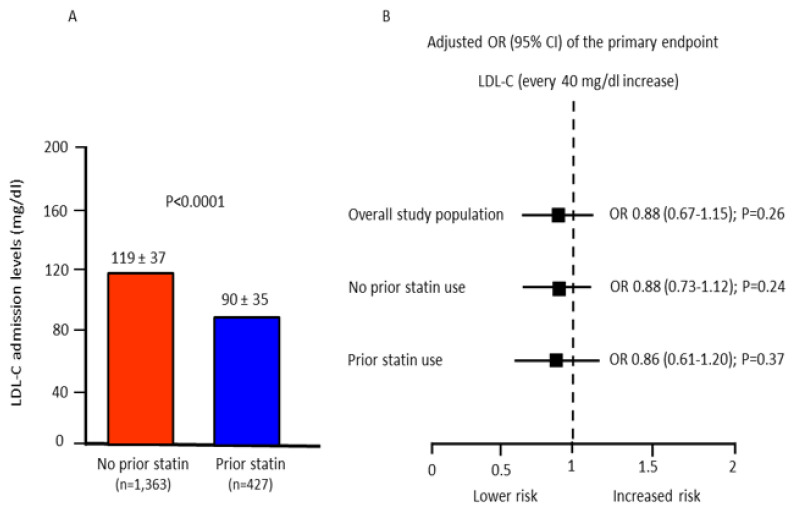
(**A**): Low-density-lipoprotein cholesterol (LDL-C) admission values in patients with or without prior statin therapy. (**B**): adjusted odds ratio (OR) and 95% confidence intervals (CI) of the primary endpoint associated with every 40 mg/dl LDL-C increase in the overall study population and in patients with or without prior statin use. Odds ratios were adjusted for age, gender, prior acute myocardial infarction, diabetes mellitus, time-to-treatment, and STEMI location (anterior vs. non-anterior).

The adjusted risk of the primary endpoint was not associated with LDL-C levels at admission both in patients with and without prior statin therapy (Figure 4).

**Figure 5:**
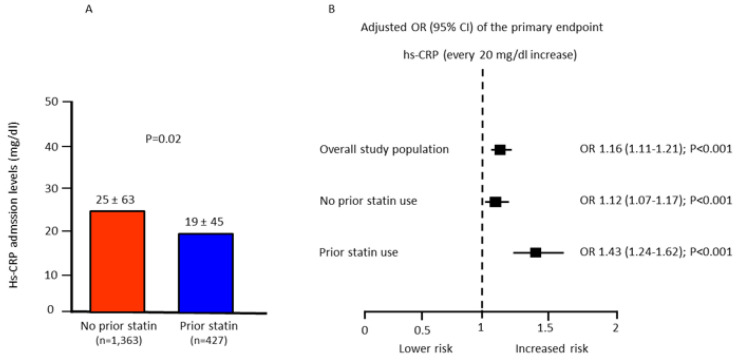
(**A**): high-sensitivity C-reactive protein (hs-CRP) admission values in patients with or without prior statin therapy. (**B**): adjusted odds ratio (OR) and 95% confidence intervals (CI) of the primary endpoint associated with every 20 mg/dL hs-CRP increase in the overall study population and in patients with or without prior statin use. Odds ratios were adjusted for age, gender, prior acute myocardial infarction, diabetes mellitus, time-to-treatment, and STEMI location (anterior vs. non-anterior).

Conversely, as high-sensitivity C-reactive protein (hs-CRP) levels at admission increased, the adjusted risk of the primary endpoint progressively raised in both groups (Figure 5).

## 4. Discussion

The results of the present study demonstrate that prior statin therapy, as defined in this study, is associated with a better in-hospital clinical outcome in patients with STEMI treated with pPCI, compared to patients without prior statin treatment. 

The data supporting the early use of statin therapy in patients presenting with acute coronary syndromes represents a critical change in the daily clinical practice of the last two decades. In particular, pre-treatment with statins in non-ST-elevation acute coronary syndrome patients consistently reduced myocardial injury and markedly improved prognosis, mainly due to the pleiotropic effects of statins [7,8,9,10,19,20,21]. On the other hand, the beneficial effects of statin pre-treatment in STEMI patients are more conflicting [14,15,22,23]. Indeed, two randomized studies did not show a positive effect of statin pre-treatment on the indexes of myocardial perfusion or infarct size in patients with STEMI undergoing pPCI [15,23]. On the other hand, Kim et al. [24] found smaller infarct size in STEMI patients randomized to statin therapy before pPCI. The assessment of the extent of the infarction was, however, performed by single-photon emission computerized tomography and therefore potentially affected by the proportion of myocardial stunning in the image evaluation. A possible explanation is that the time between statin loading and coronary reperfusion is not enough to reach an effective statin blood level. The relatively short door-to-balloon time interval and to the delayed gastrointestinal drug absorption in this clinical setting, particularly in patients with a large STEMI, low cardiac output, and systemic vasoconstriction, may all play a role [16]. Interestingly, in the positive study by Kim et al. [24], door-to-balloon time was longer than that reported in the other studies. In a recent paper, a 30% reduction in myocardial infarct size—as assessed by cardiac magnetic resonance—was observed in STEMI patients chronically treated with statins before the index hospitalization, as compared to those receiving statin at hospital admission [18]. Thus, the comparison of major clinical outcomes in STEMI patients treated with pPCI with or without statin therapy before hospital admission may be a good opportunity to investigate the potential benefits of statin pre-treatment in this acute clinical setting. In this study, we compared consecutive STEMI patients treated with pPCI with and without prior statin therapy before the index event to evaluate whether prior statin therapy results in a more favorable in-hospital clinical outcome.

The results of our study support the hypothesis of the potential cardio-protective effect of statins in patients with STEMI undergoing pPCI. In particular, despite a similar incidence of the primary endpoint between the two study groups, prior statin treatment was associated with a significant 40% lower risk of the primary endpoint, after adjustment for the known major prognostic predictors in STEMI. Indeed, statin-treated patients had a worse baseline risk profile, in terms of more frequent risk factors and prior cardiovascular events, than those without prior statin therapy. The mechanism(s) underlying this beneficial clinical effect cannot be elucidated by our data; however, some speculations may be proposed. Statins have vasculo-protective and cardio-protective properties as these drugs show antiplatelet effects and ischemic preconditioning effects; they stabilize coronary atherosclerotic plaques, improve endothelial function, and also reduce distal embolization risk during percutaneous myocardial reperfusion [25,26]. Moreover, experimental studies reported that statin pre-treatment has a cardio-protective effect only when myocardial ischemia is followed by reperfusion, mainly through the inhibition of myocardial edema [27]. This observation further supports statin’s potential to limit myocardial reperfusion injury during pPCI. Accordingly, in the Statins Evaluation in Coronary Procedures and Revascularization (SECURE-PCI) trial [28], a peri-procedural administration of a loading dose of atorvastatin (80 mg) reduced the incidence of major adverse cardiovascular events at 30 days in the subgroup of patients with acute coronary syndrome undergoing PCI but not in those treated with medical therapy only (6.0% vs. 8.2%; *p* = 0.02). In line with this recent evidence, statin therapy before hospital admission or soon prior to the invasive treatment improved coronary blood flow and reduced the no-reflow phenomenon after pPCI in STEMI patients [29,30]. Accordingly, in our study, patients on prior statin therapy had a better left ventricular function soon after pPCI, as reflected by a higher adjusted mean LVEF, compared to those without prior statins. Finally, we found that the risk of the primary endpoint was not associated with LDL-C levels at admission, regardless of prior statin therapy. Conversely, it significantly increased with increasing hs-CRP levels in both study groups. As patients on prior statin therapy have significantly lower hs-CRP values than those without statin therapy, we can speculate that statin anti-inflammatory effects may be involved. Notably, it has been demonstrated that statin therapy is able to reduce the pathway signaling of the nuclear factor k (NF-kB), lowering the circulating levels of pro-inflammatory cytokines [25,26,31,32]. Finally, it has been reported that prior statin therapy reduces the incidence of serious cardiac tachyarrhythmia, such as ventricular tachyarrhythmia/ventricular fibrillation, in patients with acute myocardial infarction undergoing percutaneous revascularization [33].

Our study has some potential clinical implications. It emphasizes an additional clinical advantage associated with long-term statin treatment in patients at high cardiovascular risk, in whom therapeutic adherence should be strongly encouraged and closely monitored. Furthermore, although there is debate on the timing of statin administration in the acute phase of STEMI, our data suggest that an immediate loading dose of statin, ideally at the time of first medical contact, may be appropriate to maximize the cardioprotective effect of this class of drugs. Future randomized studies should investigate whether prompt statin administration as soon as STEMI is suspected is associated with an improved clinical outcome.

Some limitations of this study should be mentioned. First, ours is a retrospective study; thus, no cause–effect relationship can be inferred between prior statin use and improved in-hospital clinical outcomes. Second, the type and dose of chronic statin therapy were not assessed in our study and this may have affected the study results. Finally, some specific pieces of information on procedural variables, including the extent of coronary artery disease and completeness of revascularization, pre- and post-pPCI Thrombolysis in Myocardial Infarction (TIMI) flow, and myocardial blush grade, which deserve attention when referring to outcomes in STEMI, were not available. 

In conclusion, the results of the present study demonstrated that prior statin therapy is associated with a better in-hospital clinical outcome in STEMI patients being treated with pPCI. Our preliminary findings should be confirmed by future randomized clinical trials investigating whether a strategy based on a very early statin load will improve the prognosis of STEMI patients.

## Figures and Tables

**Table 1:** Baseline characteristics of study patients.

Prior Statin Therapy			
	Yes (*n* = 427)	No (*n* = 1363)	*p* Value
Age (years)	68 ± 11	65 ± 12	<0.0001
Men, *n* (%)	344 (81%)	1010 (74%)	0.007
Body weight (kg)	76 ± 14	76 ± 15	0.65
Height (cm)	169 ± 10	169 ± 9	0.55
Diabetes mellitus, *n* (%)	127 (30%)	210 (15%)	<0.0001
Hypertension, *n* (%)	306 (72%)	723 (53%)	<0.0001
Smokers, *n* (%)	146 (34%)	584 (43%)	<0.001
Prior myocardial infarction, *n*, (%)	201 (47%)	89 (7%)	<0.0001
Prior coronary artery bypass, *n* (%)	72 (17%)	35 (3%)	<0.0001
Prior PCI, *n* (%)	186 (52%)	70 (6%)	<0.0001
Time-to-treatment (h)	5 ± 7	6 ± 5	0.72
Anterior AMI, *n* (%)	167 (39%)	612 (45%)	0.03
LVEF (%)	48 ± 11	48 ± 12	0.86
Medication before hospital admission, *n* (%)			
Aspirin	159 (37%)	244 (17%)	<0.0001
Beta-blockers	184 (51%)	192 (17%)	0.01
ACE/AR blockers	187 (52%)	291 (26%)	<0.0001
Warfarin	19 (5%)	47 (4%)	0.38
Medication during CCU stay, *n* (%)			
DAPT	427 (100%)	1363 (100%)	1
Statin	427 (100%)	1363 (96%)	1
Beta-blockers	290 (82%)	902 (82%)	0.98
ACEi/ARB	244 (69%)	725 (66%)	0.24
Laboratory values at hospital admission			
Blood glucose (mg/dL)	160 ± 61	157 ± 64	0.46
Serum creatinine (mg/dL)	1 ± 0.43	1.1 ± 1.3	0.002
eGFR (mL/min/1.73 m^2^)	74 ± 25	78 ± 24	0.02
Hemoglobin (g/dL)	13.7 ± 1.8	14.0 ± 1.9	0.004
Total cholesterol (mg/dL)	156 ± 40	185 ±43	<0.0001
HDL (mg/dL)	48± 13	43 ± 13	0.2
Triglycerides (mg/dL)	114 ±62	120 ± 71	0.3

ACEi = angiotensin-converting enzyme inhibitors; ARB = angiotensin II receptor blockers; CCU = coronary care unit; DAPT = dual antiplatelet therapy; eGFR = estimated glomerular filtration rate (MDRD equation); HDL = high-density lipoprotein; LVEF = left ventricular ejection fraction; PCI = percutaneous coronary intervention; Time-to-treatment = Time from onset of pain to primary PCI.

**Table 2:** In-hospital clinical complications.

Prior Statin Therapy			
	Yes (*n* = 427)	No (*n* = 1363)	*p* Value
Mortality, *n* (%)	10 (2.3%)	50 (3.7%)	0.18
CPR, VT, or VF, *n* (%)	52 (12%)	188 (14%)	0.39
Atrial fibrillation, *n* (%)	62 (14%)	185 (14%)	0.84
High-degree AV conduction disturbances, *n* (%)	25 (6%)	56 (5%)	0.16
Acute pulmonary edema, *n* (%)	48 (11%)	158 (12%)	0.84
Respiratory failure requiring MV, *n* (%)	15 (4.2%)	72 (6.4%)	0.11
Cardiogenic shock requiring IABP, *n* (%)	30 (7%)	129 (9.5%)	0.12
Major bleeding requiring blood transfusion, *n* (%)	13 (3%)	31 (2%)	0.37
CCU length of stay (days)	4 ± 2	4 ± 3	0.89

AV = atrio-ventricular; CCU = coronary care unit; CPR = cardiopulmonary resuscitation; IABP = intra-aortic balloon pump; MV = mechanical ventilation; VF = ventricular fibrillation; VT = ventricular tachycardia.

## Data Availability

Data supporting reported results can be requested.
